# Terahertz Photoconductivity Spectra of Electrodeposited Thin Bi Films

**DOI:** 10.3390/ma14123150

**Published:** 2021-06-08

**Authors:** Ignas Nevinskas, Zenius Mockus, Remigijus Juškėnas, Ričardas Norkus, Algirdas Selskis, Eugenijus Norkus, Arūnas Krotkus

**Affiliations:** Center for Physical Sciences and Technology, Saulėtekio al. 3, LT-10257 Vilnius, Lithuania; zenius.mockus@ftmc.lt (Z.M.); remigijus.juskenas@ftmc.lt (R.J.); ricardas.norkus@ftmc.lt (R.N.); algirdas.selskis@ftmc.lt (A.S.); eugenijus.norkus@ftmc.lt (E.N.); arunas.krotkus@ftmc.lt (A.K.)

**Keywords:** bismuth film, electrodeposition, terahertz emission, terahertz photoconductivity spectra, femtosecond laser

## Abstract

Electron dynamics in the polycrystalline bismuth films were investigated by measuring emitted terahertz (THz) radiation pulses after their photoexcitation by tunable wavelength femtosecond duration optical pulses. Bi films were grown on metallic Au, Pt, and Ag substrates by the electrodeposition method with the Triton X-100 electrolyte additive, which allowed us to obtain more uniform films with consistent grain sizes on any substrate. It was shown that THz pulses are generated due to the spatial separation of photoexcited electrons and holes diffusing from the illuminated surface at different rates. The THz photoconductivity spectra analysis has led to a conclusion that the thermalization of more mobile carriers (electrons) is dominated by the carrier–carrier scattering rather than by their interaction with the lattice.

## 1. Introduction

Bismuth is a semimetal that has many extraordinary characteristics because of its high electron energy band structure anisotropy, small electron and hole effective masses, and due to their high mobilities [1]. In addition, Bi is the first material in which the electron quantum confinement effects were demonstrated [2]. A two-dimensional (2D) carrier confinement results in the semimetal—semiconductor transition in thinner than ~30 nm Bi layers [3]; quantum confinement effects are also evidenced in Bi nanowires [4] and Bi nanocrystals [5]. Few monolayer thick Bi layers, epitaxially grown on Si substrates, were reported to have a large bulk bandgap indicating possible topological insulator properties at room temperature [6]. Due to this diversity of its physical characteristics, bismuth is sometimes considered as the key material in nanoelectronics, when quantum effects rather than doping would be exploited to mimic traditional microelectronics [7].

Furthermore, due to stability in air and negligible toxicity, Bi layers are finding significant applications in energy-related applications such as electrocatalytic CO_2_ reduction [8,9,10], N_2_ reduction [11], glucose oxidation [12], or sodium-ion storage [13]. Bi is also used as an efficient catalyst in organic synthesis reactions [14]. An overview of Bi applications in different areas of catalysis was published recently in [15]. It is worth noting that both—bulk bismuth [16] as well as bismuth film electrodes [17,18]—are used for electroanalytical purposes.

As a consequence of these new and important applications, a wider investigation of thin Bi film growth technologies is required. In this contribution, the growth of Bi layer by electrodeposition was performed. This technology appears to be one of the most suitable to grow Bi since it is a fast and cost-effective procedure to obtain large area, high-quality layers on different substrates [19,20,21,22,23]. It should be noted that according to the literature data, the electrodeposited bismuth coatings are comparatively thick for our below-mentioned intensions, i.e., coating thicknesses exceed 1 µm, while Bi grain sizes are 500 nm or larger. In this study, we developed an original electrolyte for the electrodeposition of bismuth films thinner than 1 µm (details in the Experimental Part).

Bismuth layers of various thicknesses (from 50 nm to 600 nm) were electrodeposited on an Au substrate, while on Ag and Pt substrates the deposited Bi film thicknesses were 100 nm. The structures were characterized by the X-ray diffraction (XRD) and scanning electron microscopy (SEM). Then, the Bi layers were investigated by the terahertz (THz) emission spectroscopy—the measurement of the THz radiation pulse amplitudes emitted from the Bi surfaces after their photoexcitation by femtosecond optical pulses of different wavelengths. This contact-less measurement technique that is essentially related to a high temporal resolution of the photoconductivity spectrum could provide unique information on the electrical and optical characteristics of the investigated materials [24].

## 2. Layer Growth Methods and Structural Characterization

The above-mentioned methods of bismuth electrodeposition form 1 µm or thicker coatings consisting of 500 nm or larger Bi grains. To fabricate thinner Bi films, the experimental search for proper Bi electrodeposition electrolyte was carried out. A nitrate electrolyte was developed, allowing the electrodeposition of thin (50–600 nm) Bi films.

The analytical-reagent-grade chemicals and deionized water used for solution preparation are listed in Table 1 together with the plating conditions. The electrodeposition experiments were performed in a two-electrode magnetically stirred cell by applying galvanostatic method with current density of 20 mA∙cm^−2^ (potentiostat/galvanostat Reference 600 (Gamry Instruments)). The cathodes (working electrodes) were Au, Pt sheets, and on Pt electroplated 1μm thick Ag (from cyanide bath). The anode was a Pt disc. Prior to each experiment, the Au and Pt cathodes were chemically etched in a 3:1:1 water solution of H_2_SO_4_ ~ 96 wt %, H_2_O_2_ ~ 30 wt %, and then washed with deionized water and dried with nitrogen. The Pt/Ag substrates were used as deposited without any additional pre-treatment.

**Figure 1:**
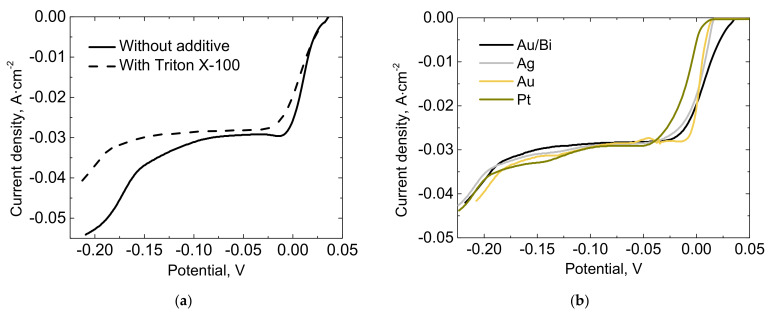
(**a**) Cathodic polarization curves of Bi electrodeposition on Bi-coated (100 nm) Au electrode from solutions with and without the additive Triton X-100. (**b**) Cathodic polarization curves of Bi electrodeposition on Bi, Au, Pt and Ag substrates from solutions with the Triton X-100 additive.

Triton X-100 is a non-ionic surfactant with a hydrophilic polyethylene oxide chain (on average it has 9.5 ethylene oxide units) and an aromatic hydrocarbon hydrophobic group, namely, 4-(1,1,3,3-tetramethylbutyl)-phenyl. In the processes of metal electrodeposition, it may act as a suppressor for three-dimensional growth of metals. It is worth noting that the surface-active substance Triton X-100 for Bi electrodeposition was applied for the first time. The influence of Triton X-100 additive on the shape and run on the cathodic polarization curve is shown in Figure 1a, whereas the influence of different substrates is shown in Figure 1b. It can be seen that the initial potential of Bi electrodeposition depends on the nature of the substrate, and it is more positive when Bi is deposited on a Bi electrode (Figure 1b). In all substrate cases, the values of the limiting currents remain approximately the same.

**Figure 2:**
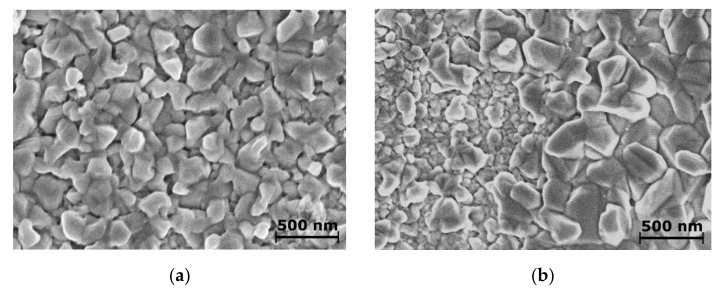
SEM images of 100 nm thick Bi coatings on Au obtained from Bi electrodeposition solution with the additive Triton X-100 (**a**) and without it (**b**).

Obviously, Triton X-100 adsorbs on Bi deposition sites suppressing crystal growth, which results in a negative polarization curve shift. The negative shift increases the Bi electrodeposition over-potential at a constant current density, which leads to an increase in nucleation density, reduction in grain sizes followed by the formation of a flat and rather smooth surface, as can be seen in Figure 2a. The morphology of Bi coatings obtained in an electroplating bath without the surface-active substance Triton X-100 differs significantly (Figure 2b). In this case, the Bi coating consists of varying sized, non-uniform crystallites. Therefore, the Triton X-100 allows us to obtain thin Bi films with uniform finely grained crystallites. A dual beam system Helios Nanolab 650 with an energy dispersive X-ray (EDX) spectrometer INCA Energy 350 and an X-Max 20 mm^2^ detector was used to estimate the thicknesses of films. The EDX data were processed with a ThinFilmID ver. 1.3.0 software, and the thicknesses of Bi layers were recalculated from 9.78 g/cm^3^ bismuth density.

**Figure 3:**
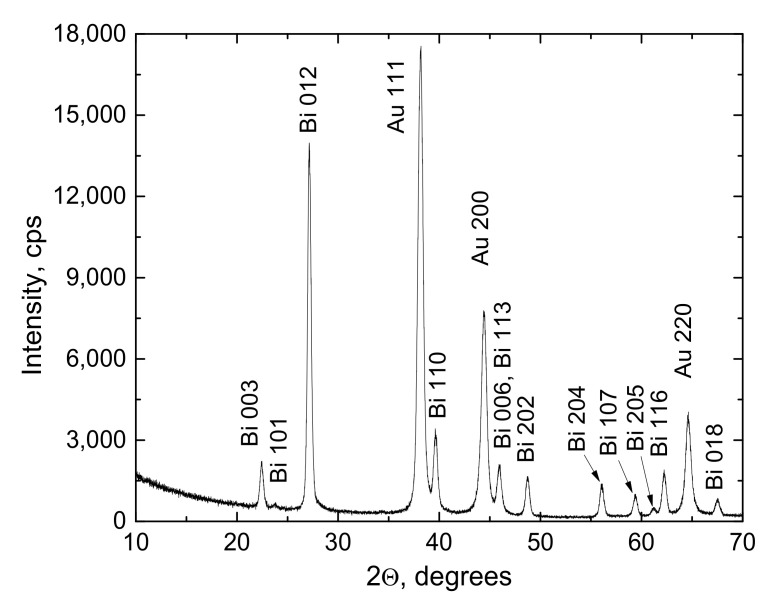
The XRD pattern of the electrodeposited 100 nm thick Bi film on Au substrate.

The XRD measurements were carried out using X-ray diffractometer SmartLab (Rigaku) equipped with a 9 kW X-ray source with a rotating Cu anode. The grazing incidence technique (also known as the out-of-plane method) was applied to obtain a high-quality XRD pattern of the electrodeposited Bi film only 100 nm in thickness. The angle between the film surface and the incident parallel beam of X-rays, ω, equaled 0.5°. The electrodeposited Bi film’s XRD pattern evidenced the film was polycrystalline of a trigonal crystalline structure (Figure 3). Even the thinnest 50 nm Bi film on gold did not present prevailing crystallographic orientation. This means that neither the XRD pattern nor SEM images of the electrodeposited Bi films are related to the substrate origin. Despite the grazing incidence technique, the XRD peaks of Au substrate were also present in the pattern. The Bi peaks coincided very well with those presented in the ICDD database card #00-044-1246. The average size of Bi crystallites calculated using Halder–Wagner method was 19.7 ± 0.7 nm.

## 3. Ultrafast Measurements

### 3.1. Experimental Setup

**Figure 4:**
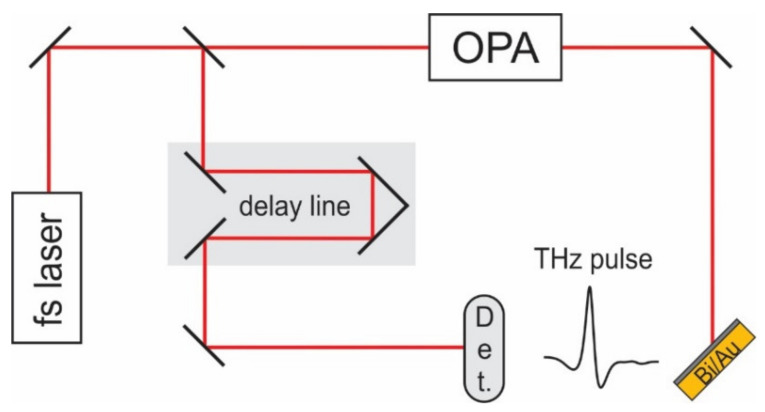
Terahertz time-domain spectroscopy setup.

The THz time-domain spectroscopy (THz-TDS) setup used for the investigations contained a Yb:KGW (λ = 1030 nm), 200 kHz pulse repetition rate PHAROS laser (Light Conversion Ltd., Vilnius, Lithuania). A small fraction of this laser beam of about 1 mW of average power was guided to illuminate the GaAsBi photoconductive antenna detector from Teravil Ltd. The rest of the beam was directed to an optical parametric amplifier (OPA) ORPHEUS (also Light Con. Ltd., Vilnius, Lithuania) with a capability to tune the wavelength of femtosecond pulses from 640 to about 2000 nm. The bismuth films were illuminated with these *p*-polarized various wavelength pulses at an angle, and the generated THz pulses were detected in the quasi-reflection direction (Figure 4).

### 3.2. Measurements

**Figure 5:**
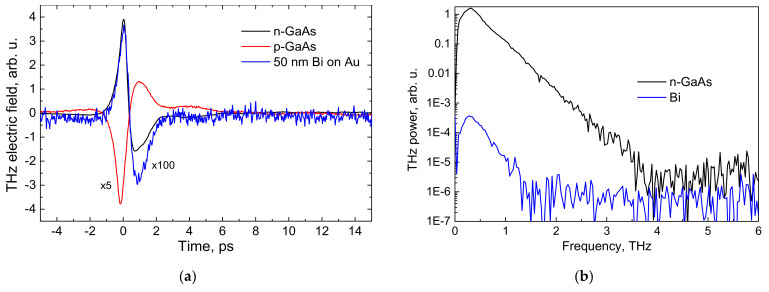
THz pulses (**a**) and their Fourier spectra (**b**) radiated from *n*-type GaAs (*n* = 2 × 10^18^ cm^−3^, black line), *p*-type GaAs (*p* = 4 × 10^18^ cm^−3^, red line) single crystals and from the surface of a 50 nm thick Bi on Au layer (blue line). The optical pulse wavelength was 780 nm, and the average power of the beam was 300 mW.

Figure 5a shows the temporal shape of a THz pulse radiated from the surface of a Bi layer grown on gold and its comparison with the pulses generated under exactly the same conditions from *n*- and *p*-type GaAs crystals. The amplitude of pulses emitted by Bi layer is about 100 times lower than those emitted by the GaAs crystals. Correspondingly narrower is the frequency spectrum of the bismuth layer emission (Figure 5b). These results are not surprising, because similar low THz emission levels from polycrystalline Bi have already been documented before [25]; however, the main reason behind this experiment is the comparison of the THz pulse polarities in all three cases. As it can be seen in Figure 5a, the THz pulse radiated from the Bi layer is of the same polarity as the pulse radiated from the *n*-type GaAs, which means that the dynamically changing electric dipole responsible for THz emission in both of these cases has the same direction. The photoexcited electrons are moving towards the bulk leaving the holes closer to the surface.

There are two main mechanisms causing the spatial separation of photoexcited electrons and holes by femtosecond optical pulses: the photocurrent, *J_ph_*, can be caused by the built-in electric field at a semiconductor surface [26] and by the different diffusion rates of both types of current carriers—the so-called photo-Dember effect [27]. In both of these cases, the THz pulse electric field amplitude, *E_THz_*, is proportional to the time derivative of the photocurrent, *E_THz_ ~ dJ_ph_/dt*. Therefore, the maximum photocurrent, which is more convenient for theoretical modeling, can be determined by integrating the THz electric field temporal dependence such as shown in Figure 5a.

## 4. Results and Discussion

**Figure 6:**
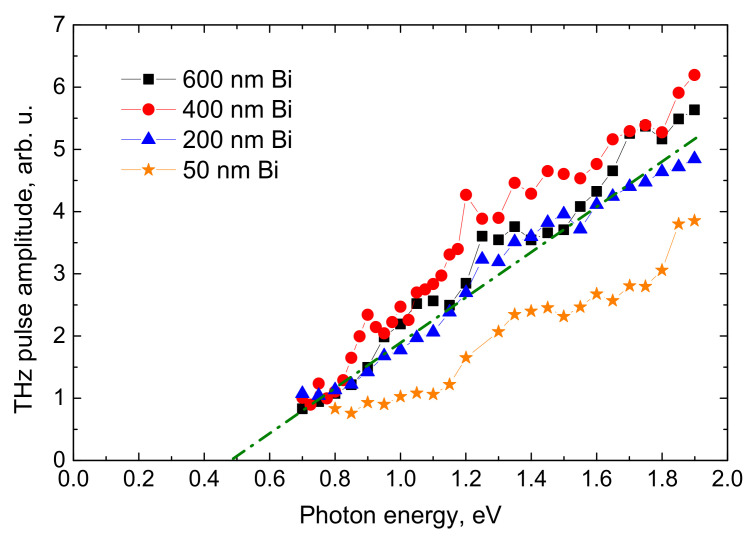
THz pulse amplitudes normalized to the number of photons in femtosecond optical pulses as a function of the photon energy. The experiment was performed on the four different thickness Bi layers grown on gold substrates.

The dependences of THz pulse amplitudes on exciting photon energy, *hν*, measured on three Bi layers of different thicknesses shown in Figure 6 are presented in a standard way used for THz excitation spectra of crystalline semiconductors [24]—with pulse amplitudes normalized to a constant photon number. It can be seen that the measured spectra are practically identical for all the investigated samples, which indicates that the physical processes leading to THz pulse emission are taking place at a rather thin layer close to the Bi/air boundary. Some reduction in the THz pulse amplitudes was observed only for thinner than 50 nm Bi films, most probably due to the incomplete coverage of the Au substrate.

In the case of direct bandgap materials, the shape of THz excitation spectra, such as shown in Figure 6, would be straightforwardly interpreted in terms of the energy bandgap *ε_g_* ≈ 0.5 eV determined from the approximated energy value at *E_THz_* = 0 and the linear THz intensity growth at higher *hν* caused by the increase of the photoelectron velocity with its increasing excess energy [24]. However, this interpretation cannot be applied to the semimetallic bismuth case. The energy bandgap in Bi is absent; there are no electron transitions between a pair of valence and conduction bands with their extrema at the same point of the Brillouin zone. The absorbed photon can excite numerous electron transitions at different points of this zone (see, e.g., [28,29]). Electron and hole excess energies for each optical transition can be different; therefore, the relation of a particular sole excess energy for each absorbed optical quantum energy becomes meaningless. Instead, it would be more sensible to analyze the electron-hole system that is heated up by a femtosecond laser pulse of a constant amount of energy but with differing its *hν* portions.

**Figure 7:**
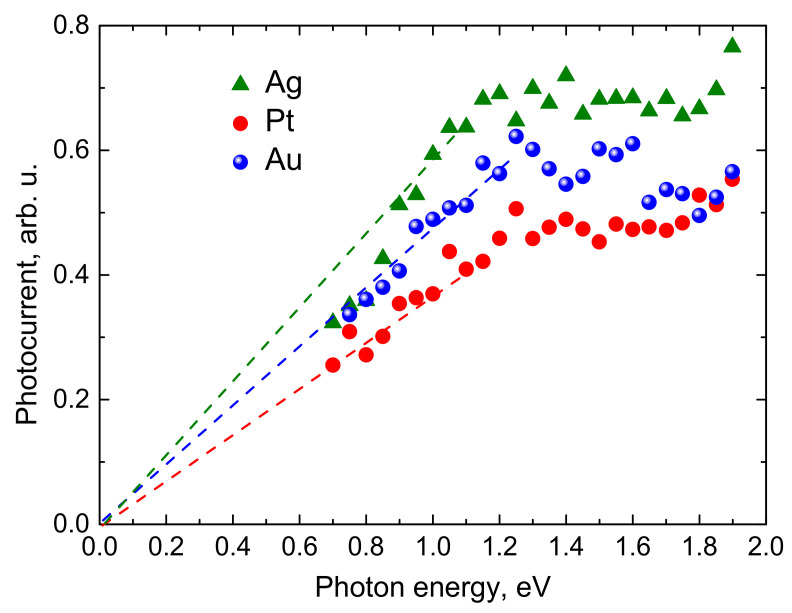
THz photocurrent as a function of the photon energy. Three 100 nm thick bismuth films on different substrates were measured.

Therefore, in Figure 7, the THz excitation spectra measured on 100 nm thick Bi layers grown on three different metallic substrates are presented as normalized to a constant optical beam intensity. The average power of the optical beam for different OPA wavelengths was changing from 200 mW to ~400 mW; in a separate experiment (not shown), it was found out that the THz signal amplitude changes nearly linearly with the optical beam intensity. Moreover, the peak values of the photocurrent transients obtained after integrating THz pulses rather than THz pulse amplitudes were plotted on those graphs. From Figure 7, it can be seen that the THz excitation spectra of polycrystalline Bi layers grown on three different substrates show only slight quantitative differences. Qualitatively, all three dependences have the same main features: a linear increase at lower photon energy range without any particular onset energy and saturation at *hν* > 1 eV.

Since the energy band bending and built-in electric fields in semi-insulating Bi are absent, the most probable cause of the ultrafast photocurrents and THz pulse emission is the photo-Dember effect. In the following, we will try to explain the obtained experimental dependences by using a simple model of this effect proposed by M. Tonouchi in [30] together with spectral characteristics of the polycrystalline bismuth optical response [31]. In general, the diffusion current at the photoexcited layer’s surface is proportional to the diffusion coefficient, *D*, and the carrier density gradient:

The polarity of THz pulses shown above (Figure 5a) evidences that electrons are more mobile carriers, thus the electron transport parameters should be inserted into the equation. It was shown in [29] that the photoexcited electrons in Bi thermalize via carrier–carrier scattering and this thermalization takes place at the femtosecond time scale; the phonon scattering being few orders of magnitude slower. One can, therefore, assume that the electrons participating in THz emission would have thermal distribution with a characteristic temperature *T_e_ ~ hν.* On the other hand, *D ~ μ_e_T_e_* and the electron mobility *μ_e_* in Bi under dominating intercarrier scattering may be described by a power function *μ_e_ ~* 1/*T_e_*^0.7^ [29]. The carrier density gradient can be roughly approximated by the ratio of the absorbed photon number and the absorption depth, *dn/dz ~ P_op_ ∙ (1 − R)/hν ∙ α*, where *P_op_* is the optical beam intensity, *R* is the reflectance, and *α* is the absorption coefficient. After inserting these expressions into Equation (1), one obtains a functional dependence of the photocurrent amplitude on photon energy:

(1) $$J_{d}\sim D\frac{dn}{dz}$$

**Figure 8:**
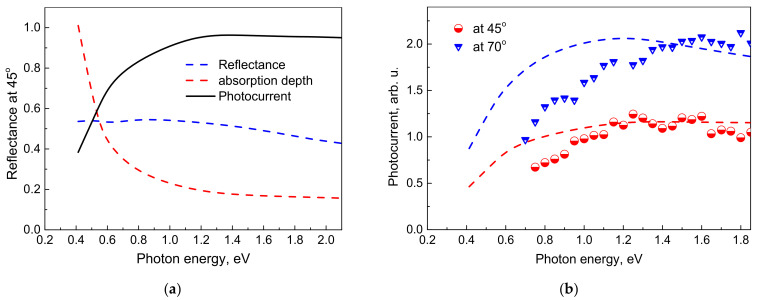
(**a**) THz photocurrent spectrum calculated using Equation (2) and the reflectance (blue line) and absorption depth (red line) spectra. (**b**) THz photocurrent spectra measured on 100 nm thick Bi on Au layer excited at different illumination angles. Points—experiments; dashed lines—calculations.

The spectral photocurrent, *J_d_*, is calculated by using Equation (2) together with spectral dependences of the absorption depth, α^−1^, and the reflectance, *R,* impinging on the Bi layer surface at a 45° angle, both taken from [31] (see Figure 8a). The calculated shape of the photocurrent spectrum closely repeats the shape of the experimental dependences shown in Figure 7. The onset of the saturation is coinciding with the spectral range, where the change of the absorption depth is also becoming slower. It evidences that THz pulse emission observed in our experiments is indeed originating from the photo-Dember effect, and that the observed THz photocurrent saturation is caused by the absorption spectrum peculiarities of the polycrystalline Bi.

(2) $$J_{d}\sim \frac{h\nu }{{\left(h\nu \right)}^{0.7}}P_{op}\frac{\left(1-R\right)}{h\nu }\alpha \;\to \;\frac{\left(1-R\right)}{h\nu^{0.7}}\alpha$$

As an additional proof of these conclusions in Figure 8b, we present the results obtained for two different angles at which the femtosecond optical pulses are impinging the Bi layer surface. Briefly, 70° is close to the Brewster angle in Bi; therefore, the enhanced light absorption is the main cause of the photocurrent increase. Theoretical curves coincide with the experimental points in the photocurrent saturation range; a significant disagreement, most probably caused by the roughness of the model, is observed at the initial parts of the spectra.

## 5. Conclusions

Polycrystalline bismuth films were electrodeposited on three different noble metals: Au, Ag, and Pt. It was found that the addition of the surface-active substance Triton X-100 significantly improves the deposition conditions and allows us to obtain 50 nm to 600 nm thick Bi films consisting of uniform finely grained crystallites. These films were investigated with femtosecond optical pulses of a tunable wavelength from 0.6 μm to 2 μm. Spectral dependences of the pulsed THz radiation emission were measured, and the photoconductivity spectra of Bi films were determined from these measurements. It was established that the photoconductivity spectrum does not depend on Bi film thickness and it is only slightly dependent on the substrate material. This led us to the conclusion that the photoconductivity effect in polycrystalline bismuth films is originating from nonequilibrium carrier diffusion and that the photoexcited electrons thermalize during THz pulse generation due to the intense carrier–carrier scattering events.

## Figures and Tables

**Table 1:** Bath composition and plating conditions for thin Bi film electrodeposition.

Chemicals	Concentration (M)
Bi(NO_3_)_3_∙5H_2_O	0.15
HNO_3_	1.5
KNO_3_	1.0
Triton X-100 C_14_H_22_O(C_2_H_4_O)_n_ *n* = 9–10	0.0015
Current density	20 mA∙cm^−2^
Bath temperature	Room temperature
Magnetic stirring	500 rpm
Electrodeposition rate	500 nm∙min^−1^
pH	~0

## Data Availability

Publicly available datasets were analyzed in this study. This data can be found here: https://github.com/Indulgence21/Bismuth-film-on-Metal.
